# Verification of inverse planning and treatment delivery for segmental IMRT

**DOI:** 10.1120/jacmp.v5i2.1975

**Published:** 2004-08-16

**Authors:** James L. Bedford, Peter J. Childs, Alan P. Warrington

**Affiliations:** ^1^ Joint Department of Physics The Institute of Cancer Research and Royal Marsden NHS Foundation Trust Downs Road Sutton Surrey SM2 5PT United Kingdom

**Keywords:** treatment planning, IMRT, verification, step‐and‐shoot, multiple static fields

## Abstract

With intensity‐modulated radiotherapy (IMRT), it is important that the inverse planning process yields the most appropriate dose distribution for the patient and that the delivered dose then corresponds to the planned dose. This paper presents methods by which the inverse planning and delivery of segmental (step‐and‐shoot) IMRT can be verified, and gives results for a typical treatment planning system (Pinnacle^3^ v6.2b, Philips Radiation Oncology Systems, Milpitas, CA). Inverse planning was assessed by observing the reduction in objective function as fields were successively added to three‐field prostate, esophagus, and thyroid plans. The ability of the treatment planning system to calculate dose for a segmented field was examined by creating a stepped field with five successively narrowing segments. The complete planning process was then investigated by using two orthogonal IMRT fields to create a homogeneous dose distribution in a cubic water phantom. Finally, a clinical situation was simulated by creating a five‐field segmental IMRT plan for a lung target in an anthropomorphic phantom. A conformal plan was also compared for context. Addition of fields to inverse plans generally resulted in a reduction of objective function, indicating consistency of inverse planning solutions. Planned dose for fields with stepped intensity agreed with ionization chamber measurements to within 5%. For orthogonal fields, planned dose distributions agreed well with dose measured using film and agreed with ionization chamber measurements to within 3%. For the anthropomorphic phantom, the standard deviation of difference between planned and measured dose was 4%. Although no consensus has yet been reached on what constitutes an acceptable IMRT plan, these results indicate that step‐and‐shoot IMRT can be planned and delivered using the system described with comparable accuracy to a standard conformal treatment.

PACS numbers: 87.53.Dq, 87.53.Kn, 87.53.Mr, 87.53.Tf, 87.53.Xd

## I. INTRODUCTION

As the use of intensity‐modulated radiotherapy (IMRT) becomes more widespread in clinical practice, it is important to be able to verify that the planned and delivered dose distributions are appropriate. A variety of inverse planning techniques and delivery methods are in use,^(1)^ and each of these presents its own challenges in terms of obtaining the optimal treatment plan for the patient and ensuring that the delivered dose distribution closely matches the planned distribution. The majority of IMRT is inverse planned, and it is difficult to ensure that the treatment plan proposed by the inverse planning algorithm is optimal for the patient being treated. This is because the optimum usually involves a compromise between the dose to the planning target volume and the various critical structures. This compromise is often handled by means of importance factors in the inverse planning algorithm, and these are hard to determine.^(2)^ ^,^ ^(3)^ Furthermore, if the optimization algorithm consists of a gradient descent method or a stochastic search, there is no guarantee that the optimization algorithm reaches a global optimum. In the case of the gradient descent methods, the algorithm may not be sophisticated enough to overcome local minima, and with random search methods, the algorithm may not be run for enough iterations to find the global minimum. In particular, these problems are a possibility in the commercial systems, where the need to provide a solution in a clinically feasible length of time is often given priority over the generation of a truly optimal dose distribution. However, there have been virtually no reports in the literature concerning the verification of the inverse planning process itself. Therefore, it is important that this component of the IMRT treatment process is addressed.

In contrast, the treatment delivery part of the process has been well studied.^(4)^ Attention has been directed to reproduction of patient treatments on verification phantoms.^(5)^ ^,^ ^(6)^ This type of verification has in turn prompted attention to the measurement devices used, such as ionization chambers^(7)^ and MOSFET.^(8)^ Specialized solutions have also been proposed, such as beam imaging systems^(9)^ and diode arrays.^(10)^ Verification film, appropriately handled, is ideally suited to validating dose distributions, and this has given rise to careful evaluation of film characteristics for different field conditions.^(11)^ More novel approaches, such as polyacrylamide gel^(12)^ and Fricke gel,^(13)^ are also under investigation, while portal imaging has an important role to play.^(14)^ ^,^ ^(15)^ Others have developed verification methods using independent dose calculations rather than measurements.^(16)^ All of these verification methods have intrinsic uncertainties, and it is therefore important to understand the limitations of the measurement technique as well as the possible inaccuracy of the dose distribution being verified.

A recently published guidance document provides a framework for the clinical implementation of IMRT, including some of these issues.^(17)^ This document provides overall guidance for the operation of an IMRT treatment scheme while avoiding prescribing specific procedures. The details of the precise methods used in the verification of IMRT treatment planning and delivery are still subject to some discussion. In particular, in the verification of dose distributions, it is helpful to be aware of the fundamental capability of the dose calculation algorithm used. Any differences between calculations and measurements in complex treatments can then be interpreted in terms of the differences found—and understood—in simple situations. A set of simple tests is therefore invaluable for assessing the performance of a treatment planning system for IMRT techniques. These tests can form the basis for a more extensive site‐specific or patient‐specific verification program. Such tests are described below.

This paper focuses on the verification of IMRT inverse planning and delivery. A series of tests is presented to assess, first, the reliability of an inverse planning algorithm and second, the accuracy of the resulting calculated dose distributions. For delivery, the segmental (step‐and‐shoot) method is used because this has become firmly established as a practical and reliable means of IMRT treatment. In this method, the linear accelerator automatically delivers a number of subfields at a fixed gantry angle, the subfields being delineated by a multileaf collimator, which is reshaped between irradiations. Although results are presented for the Pinnacle^3^ treatment planning system (v6.2b, Philips Radiation Oncology Systems, Milpitas, CA), the methods are applicable to any IMRT treatment planning system. The tests described in this paper are not intended to provide an exhaustive list of checks to be carried out when commissioning an IMRT program, but rather to serve as a basis for understanding the fundamental capability of the treatment planning system, around which a more comprehensive procedure can be established.

## II. METHODS

### A. Inverse planning

The optimization algorithm within Pinnacle^3^ requires the clinical goals to be expressed in terms of minimum dose, maximum dose, dose‐volume points, or uniform dose. These can be specified as either objectives or hard constraints. For each objective or constraint, an objective function is used to evaluate how well this objective or constraint is met during the optimization.^(18)^ The relative importance of each clinical requirement is specified using a weighting factor which the user supplies. The sum of the individual objective functions, denoted the Composite Objective Function (COF), provides an overall indicator of optimality of the treatment plan. During the optimization, the COF is minimized using Stanford Systems Optimization Laboratory's Nonlinear Programming method (NPSOL), an algorithm for solving constrained nonlinear optimization problems.^(19)^

To test whether the algorithm provided truly optimal results was not possible because this requires an exhaustive search over all possible parameter combinations, which would have been prohibitively time‐consuming. However, the *consistency* of the algorithm's performance was evaluated by comparing plans with differing numbers of fields. The principle of the test was that by starting with a few fields and then subsequently adding additional fields, the quality of the plan should always increase. This was because the algorithm should have been able to reduce the intensity of the additional fields if they were unfavorable. This approach evaluated whether the algorithm was searching the space of beam parameters sufficiently and producing globally optimal solutions rather than local optima. Note that this method could be considered the practical analog of the framework developed by Crooks et al.^(20)^ for evaluating the effect of adding fields to a treatment plan.

Inverse plans were created for three different tumor sites: prostate, esophagus, and thyroid, which represented distinctly varying tumor shapes.^(21)^ Plans were created using three fields (3F), with gantry angles 0°, 120°, and 240°; five fields (5F), with 3F gantry angles plus 40° and 320°; seven fields (7F), with 5F gantry angles plus 80° and 280°; and nine fields (9F), with 7F gantry angles plus 160° and 200°. All of these plans were coplanar. Dose‐volume objectives and importance factors were assigned to the planning target volumes and critical structures, according to Table 1. Note that the importance factors were relative weights for each structure and were allowed to vary between 0.1 and 100. Thus, for the thyroid plan, it was important that the spinal cord dose was below tolerance, so this was assigned a high importance factor. Uniform dose to the planning target volume (PTV) was desirable, but only after the spinal cord constraint had been met, so the PTV was assigned a low weight. Since the importance factors were relative figures only, variation in magnitude between plans was of no effect, provided that the *relative* weights of the different structures were maintained. Consequently, the importance factor for the PTV varied considerably for the different plans, depending upon the relative importance of the other structures in each of the plans.

**Table 1:** Clinical objectives for the three cases used to assess consistency of inverse planning

Plan	Structure	Objective	Importance factor
esophagus	PTV	uniform dose 55 Gy	100
	spinal cord	maximum dose 45 Gy	10
	left lung	$V_{18}$ ^a^ less than 10%	1
	right lung	$V_{18}$ less than 10%	1
prostate	PTV	uniform dose 74 Gy	20
	rectum	$V_{60}<20\%$	10
		$V_{40}<30\%$	5
	bladder	$V_{60}<20\%$	1
	left femoral head	$V_{52}<10\%$	2
	right femoral head	$V_{52}<10\%$	2
thyroid	PTV	uniform dose 60 Gy	0.1
	spinal cord	maximum dose 45 Gy	100

^a^ This notation denotes volume of structure irradiated to 18 Gy and so on.

The optimization was run for 100 iterations, and the effectiveness of each plan was measured using the COF available within Pinnacle^3^, which reduced in value as the treatment objectives were met more precisely. Optimization took around 10 min on a 900 MHz SunBlade 2000. After an initial run with the starting beam weights all equal, the beams were reset and the starting beam weights were perturbed by 10% (e.g., 33%, 33%, 33% became 43%, 23%, 33% for a 3F plan). The run was then repeated. This was to assess the dependence of the algorithm on the starting conditions and to indicate whether the solution was a global minimum.

### B. Segmentation

Pinnacle^3^ provided a two‐stage IMRT algorithm, whereby optimized fluence profiles were generated for some combination of beam energy and orientation in the first phase, and these were then segmented into step‐and‐shoot fields in the second phase. Having tested the accuracy of the optimization within Pinnacle^3^, the next step was therefore to verify the performance of the segmentation algorithm. This algorithm converted the fluence profiles resulting from optimization into deliverable segments using K‐means clustering.^(22)^ ^,^ ^(23)^ Both segment shapes and weights were selected by this process. The principal parameters controlling the algorithm were (1) the error tolerance, which governed how closely the algorithm attempted to replicate the optimized fluence profiles using segmental delivery, a small value tending to give many segments so as to accurately match the optimized profiles; (2) the minimum equivalent square for the collimator settings; and (3) the minimum area actually exposed by the multileaf collimator (MLC) leaves. Note that although the error tolerance introduced a difference between the optimized dose distributions and the actual deliverable dose distributions, the final dose calculation was performed for the segmented fields. The final dose calculation therefore included the effects of the delivery device, such as MLC leaf transmission. (Interleaf leakage and tongue‐and‐groove effect were not explicitly modeled by this version of the treatment planning system, but are included in a later version of the software.) Thus, the planned dose distribution was expected to equate to the measured dose distribution, regardless of the parameters used for segmentation.

The segments were designed to be deliverable with an SL15 linear accelerator (Elekta Oncology Systems, Crawley, U.K.), which was selected within the treatment planning system and used for the verifications. The inverse plans generated above were segmented using several combinations of parameters, and the number, shape, and weights of the resulting beam segments were assessed. The goal of this exercise was to ensure that the segmentation process provided a feasible number of segments for a limited sequencing error and hence a good quality plan. The segments were required to have a shape and size (typically greater than 3 cm equivalent square) that allowed them to be delivered with confidence in the dosimetry. The weights of the segments were required to be sufficiently high so that the segments were worth delivering [e.g., greater than 5 monitor units (MUs)]. Furthermore, the segments were to be reasonably logical in their sequence, with each segment providing a boost to the previous one, and with not too many abutting segments.

### C. Dosimetry of segmented fields

**Figure 1:**
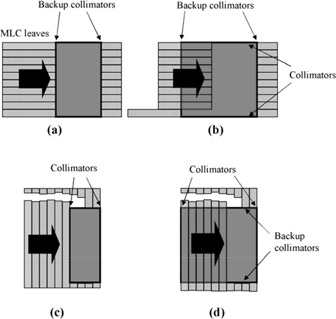
Field configurations used for testing the dose calculation for segmented fields. In each case, there are five segments. In (a) and (b), modulation is parallel to the direction of MLC leaf motion, whereas in (c) and (d), modulation is perpendicular to the direction of MLC leaf motion. In (c) and (d), pairs of leaves are successively drawn completely across the field so as to narrow its width in the direction of the arrow. In (a) and (c), the segments are defined by both MLC leaves and collimators, whereas in (b) and (d), the segments are defined by MLC leaves only.

Further tests were performed to assess the accuracy of dose calculation for segmented fields. The dose calculation provided within Pinnacle^3^ was based upon a predominantly physical but partly empirical model of the accelerator head, and a collapsed cone convolution method for determining phantom scatter.^(24)^ ^,^ ^(25)^ A 6 MV 10 cm×10 cm field was manually segmented with the treatment planning system and delivered using an Elekta linear accelerator. Each field consisted of five segments, the first consisting of an open field and the subsequent four successively decreasing in width by 2 cm, so that the final segment was 2 cm×10 cm. This was performed either by closing the MLC leaves by 2 cm or by completely closing off two leaves, so that the direction of the fluence gradient was either parallel or perpendicular to the direction of leaf motion (Fig. 1). For each of these two situations, the collimators were either reduced in width in accord with the MLC position or left open at 10 cm×10 cm for all segments. (Note that the former is the normal mode of operation for the Elekta linear accelerator, but the latter is permitted and may approximately occur during an IMRT delivery if a large number of MLC leaves are closed and only several left open.) For the situation where the dose gradient was perpendicular to the direction of leaf motion and the collimators were aligned with the MLC leaves [see Fig. 1(c)], the field was delivered 3 cm off‐axis, so that the closing X‐collimator was not required to move over the central axis. The two directions of fluence gradient and the two possible collimator strategies yielded four different field configurations (Fig. 1). This method tested the accuracy of Pinnacle^3^ in calculating elongated and off‐axis fields, as well as the accuracy in calculating penumbra due to the leaf ends and sides. It also tested Pinnacle^3^'s ability to correctly calculate the transmission through the MLC and the collimators. The method also evaluated the calculation of head scatter for various combinations of MLC and collimator position.

The test fields were planned and delivered with a source‐to‐surface distance of 100 cm, and the dose was measured at 5 cm depth in a water phantom. Measurements were made using a 0.6 cm^3^ ionization chamber (Saint Gobain Crystals and Detectors, Reading, U.K.) positioned centrally with respect to the 10 cm field width and at –4.2, –2.1, 0, 2.1, and 4.2 cm off‐axis with respect to the other field dimension, thereby being positioned at the center of each fluence “step.” The axis of the chamber was positioned along the fluence steps so that there was virtually no gradient along the length of the chamber (apart from negligible beam flatness effects orthogonal to the direction of fluence modulation) and minimal dose gradient across the width of the chamber. The dose readings from the ionization chamber were therefore taken to be representative of the dose at the center of the chamber. An EDR2 film (Eastman Kodak, Rochester, NY) was also used to measure the dose distribution under 3.8 cm of Perspex. In this case, the phantom within Pinnacle^3^ was specified to have a density of $1.15\;g\cdot {\text{cm}}^{-3}$. For both the ionization chamber measurements and film measurements, 200 MUs were used.

The film was calibrated under 3.8 cm of Perspex using up to 300 MUs with 5 cm×5 cm fields. The calibration fields had the same energy as the experimental fields, and the calibration film was oriented orthogonally to the field axis to match the orientation used in the main experiment. Four fields were delivered to the four corners of a single film, creating four points in the calibration curve. The optical density of the region in the center of the film was used to subtract background from the exposures. No shielding was used to protect the parts of the film not being directly irradiated from receiving background dose. However, it was found that the density of the parts of the film that were not deliberately irradiated was similar whether one or four irradiations were made on the single film. Most of the background optical density appeared to result from the inherent density of the film substrate itself. The calibration films were processed at the same time as the measurement films, so that the calibration procedure included the effects of film processing. The films were subsequently digitized using a VXR‐12 Plus film digitizer (Vidar Systems Corporation, Herndon, VA), using a resolution of 75 dots per inch.

### D. Orthogonal fields

This test was designed to verify the complete IMRT planning process for a simple, easily predictable case. Two orthogonal 6 MV fields, one with gantry angle 0° and the other with gantry angle 90°, were planned and delivered on a 30 cm×30 cm×30 cm solid water phantom (Radiation Measurements Inc., Middleton, WI). A 10 cm×10 cm×10 cm PTV was created at the center of the phantom, and the isocenter was designated as the center of this PTV. Inverse planning was used to create a homogeneous dose distribution across the PTV. This could be conveniently compared with the dose distribution provided by equally weighted 45° wedge beams. The IMRT fields were then segmented using an error tolerance of 3%, a minimum equivalent square of 3 cm, and a minimum segment area of 9 cm^2^.

The resulting plan was verified in the phantom by making ionization chamber measurements (0.6 cm^3^) for the two fields separately on their respective central axes at 5 cm deep and at 15 cm deep (i.e., at the isocenter). Care was taken to ensure that the fluences around the central axes of the fields were uniform, so that the ionization chamber readings could be taken as representative of the dose at the central axes. If the fluence had not been uniform, it would have been necessary to compare the chamber reading with the calculated mean dose over the chamber volume. An orthogonal EDR2 verification film was also exposed at 5 cm deep for the two fields separately. The complete dose distribution due to both fields was assessed by exposing transverse EDR2 films at the central transverse plane and 2 cm and 5 cm toward and away from the gantry, with respect to the central transverse plane. Film calibration was carried out orthogonal to the exposing 5 cm×5 cm fields at 5 cm depth in solid water, with a source‐to‐surface distance of 100 cm and using up to 400 MUs. This test therefore checked the quality of inverse planning, the segmentation performance, and the accuracy of dose calculation.

### E. Anthropomorphic phantom

A more realistic assessment of the complete IMRT planning process was performed for a lung PTV in an anthropomorphic phantom. The PTV represented a mediastinal tumor based upon that of a previous patient. The treatment was inverse planned using five equally spaced 6 MV coplanar fields, segmented using similar parameters to those used for the orthogonal fields test, and delivered to the phantom. Lithium fluoride thermoluminescent dosimeters (TLD) were used to check the dose distribution. A dose of 1 Gy was prescribed to the isocenter for this verification to minimize supralinearity effects in the TLD. Transaxial EDR2 verification films were also used to assess the dose distribution, a prescribed dose of 2 Gy at the isocenter being used for this part of the investigation. The films were exposed in their envelopes; to allow the films to fit around the phantom rods, two holes were cut out of each film and made light‐tight using tape. The nuts on the end of the phantom rods were then tightened to compress the phantom and to expel as much air as possible from the film envelopes. The optical density distribution was related to the isocenter by marking the intersection of the room lasers with the film envelopes and then pricking the films. To relate the results to established practice, the same treatment was planned using five nonmodulated conformal fields with the same directions as the IMRT fields. This treatment was then delivered to the phantom and assessed using the same film technique as with IMRT.

## III. RESULTS

### A. Inverse planning

**Figure 2:**
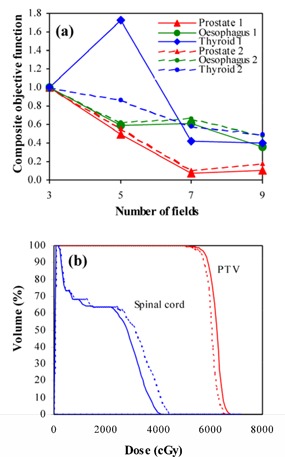
(a) Variation of composite objective function with different numbers of fields for equal starting beam weights (large symbols and solid lines) and with starting beam weights perturbed by 10% (small symbols and dashed lines). (b) Dose‐volume histograms for the five‐field thyroid plan, with equal starting beam weights (solid lines) and starting beam weights perturbed by 10% (dashed lines).

The variation in the composite objective function with increasing numbers of fields is shown in Fig. 2(a). In general, addition of fields produces a reduction in the objective function, reflecting the improvement in plan quality. The COF is also relatively unaffected by the starting beam weights, which further indicates that a global optimum is being found. Several exceptions to this trend are observed, suggesting that in these cases, the optimization terminates in a local optimum, rather than the true global optimum. The most notable case is the 5F thyroid plan, for which the COF is higher with 5F than with 3F or 7F. This situation is remedied when the starting beam weights are perturbed. The clinical difference between the result for each of the two different starting conditions with the 5F thyroid plan is shown in Fig. 2(b). With the starting weights set equal, the spinal cord dose is lower, but this does not reduce the COF because the objective simply stipulates that the maximum spinal cord dose should be less than 45 Gy. Meanwhile, the PTV dose is not so close to 60 Gy, so the COF is higher.

### B. Segmentation

By selecting an allowed dosimetric difference of 3% between the ideal inverse plans and the segmental plans, around 5 to 15 segments per field are typically generated. These are deliverable with an Elekta linear accelerator without editing. In some cases, however, minor editing can produce a plan with the same efficacy but with slightly fewer segments.

### C. Dosimetry of segmented fields

**Figure 3:**
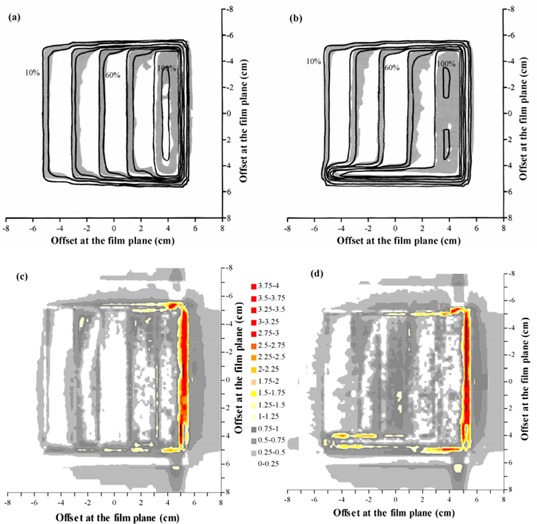
Comparison of calculated dose and dose measured using film for a single segmented field, with the modulation parallel to the direction of leaf motion. (a) Superposition of calculated (lines) and measured (gray scale) dose distributions, with the field defined by both MLC leaves and collimators. (b) Superposition of calculated (lines) and measured (gray scale) dose distributions, with the field defined by MLC leaves only. (c) Gamma distribution for criteria of 4% and 4 mm, with the field defined by both MLC leaves and collimators. (d) Gamma distribution for criteria of 4% and 4 mm, with the field defined by MLC leaves only. In (a) and (b), isodoses are at intervals of 10%, relative to the dose at the center of the high‐dose step. In (c) and (d), gray scale denotes those areas where the acceptance criteria are met.

**Figure 4:**
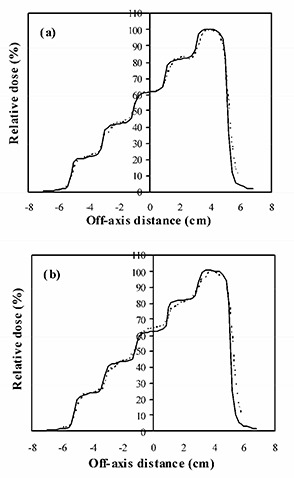
Calculated dose profile (solid lines) and dose profile measured using film (broken lines) for a single segmented field, with the modulation parallel to the direction of leaf motion. (a) Field defined by both MLC leaves and collimators. (b) Field defined by MLC leaves only.

A beam profile measured for a manually segmented field with dose gradient in the direction of leaf motion [see Fig. 1(a)] is shown in Fig. 3(a). In this case, the collimators are aligned with the MLC leaves as they successively close in. A gamma distribution^(^ ^26^ ^,^ ^27^ ^)^ for this situation is shown in Fig. 3(c), and a section along the central axis of this dose distribution is shown in Fig. 4(a)). The dose distributions in these figures are normalized to the center of the high‐dose step. The measured and calculated dose distributions are generally in good agreement, with the exceptions that the gradients of the dose steps are overestimated by Pinnacle^3^. Figs. 3(b)), 3(d), and 4(b) show the corresponding situation with the collimators left open, while the leaves close in. The agreement is not as good, with Pinnacle^3^ showing a higher or lower dose on some of the steps compared to film.

The ionization chamber measurements are shown in Table 2 for the cases where the collimators either close in with the MLC leaves or remain open. For the case when the collimators close in, the measured doses are generally within 5% of the planned doses. Closer agreement of within 2% is seen with the case where the collimators remain open.

**Table 2:** Ionization chamber measurements for verification of a single segmented field, with the dose gradient parallel to the direction of leaf motion

	Collimators following MLC		Collimators open	
Position (cm)^a^	Calc. dose (cGy)	Difference (%)^b^	Calc. dose (cGy)	Difference (%)^b^
–4.2	163	–4.4 (–2.8)	167	1.2 (0.8)
–2.1	134	–4.7 (–3.5)	138	–0.7 (–0.6)
0	101	–3.3 (–3.3)	105	–0.1 (–0.1)
2.1	69	–2.7 (–3.9)	73	–0.1 (–0.2)
4.2	36	–1.5 (–4.3)	40	0.6 (1.6)

^a^ Positions are relative to the central axis of the field, at 5 cm depth.

^b^ Dose differences are shown as a percentage of the central axis dose and as a percentage of local dose in parentheses.

**Figure 5:**
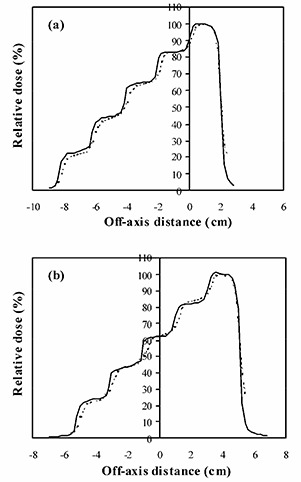
Calculated dose profile (solid lines) and dose profile measured using film (broken lines) for a single segmented field, with the modulation perpendicular to the direction of leaf motion. (a) Field defined by both MLC leaves and collimators. (b) Field defined by MLC leaves only.

Fig. 5 and Table 3 show the film and ionization chamber measurements, respectively, for the situation where the leaves are closed over two at a time, so that the dose gradient is perpendicular to the direction of leaf motion [see Figs. 1(c)) and 1(d)]. The film measurements show that Pinnacle^3^ overestimates the gradient of the dose steps slightly, but the overall agreement is good.

**Table 3:** Ionization chamber measurements for verification of a single segmented field, with the dose gradient perpendicular to the direction of leaf motion

	Collimators following MLC		Collimators open	
Position (cm)^a^	Calc. dose (cGy)	Difference (%)^b^	Calc. dose (cGy)	Difference (%)^b^
–4.2	159	–3.4 (–2.2)	167	2.3 (1.5)
–2.1	134	–1.9 (–1.5)	138	–0.1 (–0.1)
0	104	–0.5 (–0.5)	105	0.6 (0.6)
2.1	71	0.4 (0.6)	73	0.7 (1.1)
4.2	37	0.5 (1.4)	40	1.8 (5.1)

^a^ Positions are relative to the central axis of the field, at 5 cm depth.

^b^ Dose differences are shown as a percentage of the central axis dose and as a percentage of local dose in parentheses.

The ionization chamber measurements show an agreement between Pinnacle^3^ and measurements of better than 4% for the collimators closing in and 3% for the collimators remaining open.

### D. Orthogonal fields

**Figure 6:**
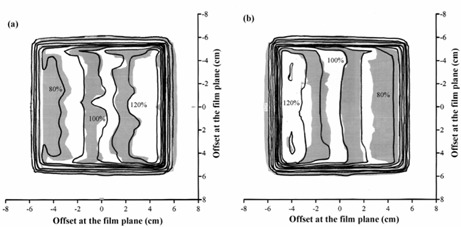
Calculated dose (lines) and dose measured using film (gray scale) for (a) the field with gantry angle 0° and (b) the field with gantry angle 90°, in an IMRT plan consisting of orthogonal fields. Isodoses are at intervals of 10%, relative to the central axis dose.

**Figure 7:**
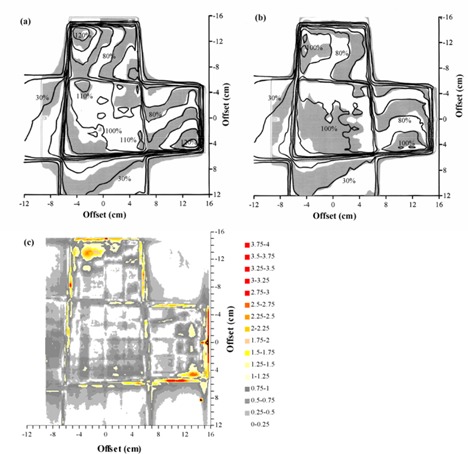
Comparison of calculated dose and dose measured using film for an IMRT plan consisting of orthogonal fields. (a) Superposition of calculated (lines) and measured (gray scale) dose distributions for a transaxial plane at the isocenter. (b) Superposition of calculated (lines) and measured (gray scale) dose distributions for a transaxial plane 5 cm farther away from the gantry than the isocenter. (c) Gamma distribution for criteria of 4% and 4 mm, for a transaxial plane 5 cm farther away from the gantry than the isocenter. In (a) and (b), isodoses are at intervals of 10%, relative to the intersection of the beam axes. In (c), gray scale indicates those areas where the acceptance criteria are met.

The dose profiles as calculated by Pinnacle^3^ and measured using film are shown in Fig. 6 for the fields at gantry angle 0° and gantry angle 90° delivered separately to the cubic solid water. The differences are to within 4%, with the larger distances between isodoses occurring in regions of low dose‐gradient. Calculated and measured dose profiles are shown in Fig. 7 for the central transverse plane and a plane 5 cm farther away from the gantry. A gamma evaluation is also shown. Again, the agreement between the isodoses is good, with larger discrepancies occurring in regions of low dose‐gradient, where the dosimetric difference is not significant. The ionization chamber measurements are given in Table 4 at 5 cm and 15 cm depths. The agreement of these ionization chamber measurements with Pinnacle^3^ is excellent, the largest difference being 2.4%.

**Table 4:** Ionization chamber measurements for verification of a pair of orthogonal fields

Field	Depth (cm)	Calc. Dose (cGy)	Difference (%)
gantry angle 0°	5	169	–0.8
gantry angle 0°	15	100	0.9
gantry angle 90°	5	167	–1.6
gantry angle 90°	15	100	2.4

### E. Anthropomorphic phantom

**Figure 8:**
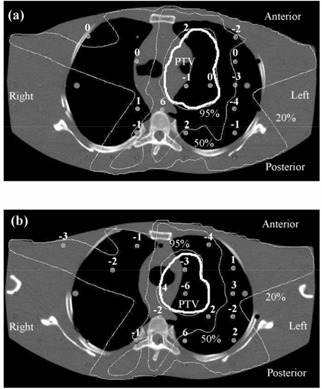
Verification of a lung IMRT plan using TLD, showing transaxial planes (a) 1 cm inferior and (b) 1.5 cm superior to the isocentric plane. The dose differences represent the planned dose in relation to the delivered dose, expressed as a percentage of the isocentric dose. Isodoses are normalized to the isocenter.

**Figure 9:**
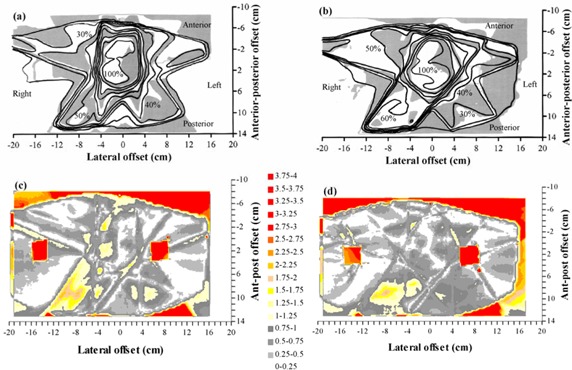
Comparison of calculated dose and dose measured using film in a transaxial plane 0.4 cm superior to the isocenter for a lung IMRT plan. (a) Superposition of calculated (lines) and measured (gray scale) dose distributions for an IMRT plan. (b) Superposition of calculated (lines) and measured (gray scale) dose distributions for the corresponding conformal plan with the same beam orientations. (c) Gamma distribution for criteria of 4% and 4 mm, for an IMRT plan. (d) Gamma distribution for criteria of 4% and 4 mm, for the corresponding conformal plan with the same beam orientations. In (a) and (b), isodoses are at intervals of 10%, relative to the intersection of the beam axes. In (c) and (d), gray scale indicates those areas where the acceptance criteria are met.

The TLD measurements in the anthropomorphic phantom are summarized in Fig. 8. Overall, the mean difference between calculated and measured doses is –0.8% (measurements higher), and the standard deviation of the difference is 3.9%. This indicates that there is no systematic difference between planned and measured doses and that the majority of measured point doses are within 4% of the corresponding planned doses. The standard deviation of 10 TLDs irradiated uniformly for calibration is 2.0%, giving an estimate of the reproducibility of the measurements. The results of the film measurements near to the central transverse plane are shown in Fig. 9(a). Fig. 9(b) shows the film results for a conformal treatment on the same PTV, giving an indication of how the IMRT verification compares with verification of a conformal plan. Note that Pinnacle^3^ has been already commissioned for these conformal techniques.^(28)^ Gamma evaluations for both IMRT and conformal plans are shown in Figs. 9(c) and 9(d), respectively. The agreement between planned and measured doses for the IMRT technique is similar to that for the conformal technique.

## IV. DISCUSSION

It is important to be confident that the inverse planning scheme used for IMRT is producing consistent and reliable solutions. The tests conducted in this study show that in most cases, the composite objective function decreases with increasing number of fields. This indicates that the inverse planning algorithm is finding a global minimum to the planning problem and producing consistent solutions. These results are in accord with the work of Söderström and Brahme^(29)^ and Stein et al.^(30)^ In several instances, Pinnacle^3^ fails to provide a sufficiently low final value of composite objective function. This indicates that it may be finding a local minimum. The inverse planning algorithm within Pinnacle^3^ considers the relatively complicated form of the objective function, taking into account both objectives and hard constraints, and locally approximates it to a quadratic objective function.^(18)^ This facilitates the use of a fast gradient algorithm, which allows the production of inverse solutions within a few minutes while taking dose‐volume objectives and constraints into account. The gradient algorithm appears to find the global minimum in most, but not all, cases.

The segmentation phase of the inverse planning has also been found to perform well in conjunction with the Elekta linear accelerators used at this center. Having specified to the planning system the physical constraints of the accelerator, the segmentation then provides solutions in accord with these constraints. In general, it is found that up to 15 segments per beam are acceptable, and this can be achieved with an error tolerance within Pinnacle^3^ of 3% to 4% and a minimum equivalent square of 2 cm to 3 cm.

The measured dosimetry of the segmented fields is in adequate agreement with the calculated dosimetry. The dose profiles measured with film are generally more rounded than the calculated profiles. Although a median filter has been applied to the measured data, this filtering operates on a much smaller scale than the steps in the dose distribution and cannot account for the rounding of the steps. The rounding must therefore be due to the treatment planning system not fully taking into account the rounded ends of the MLC leaves. (This effect has been taken into account in a later version of Pinnacle^3^.) With the ionization chamber measurements, some of the differences may be due to the uncertainty in the measurements and to the inherent limitations in the beam model used. No attempt has been made to remodel or fine‐tune the beam models used with conventional planning for IMRT purposes. This is to ensure that the existing models can function seamlessly with IMRT. The output factor calculation within Pinnacle^3^, which is based on equivalent square, is possibly responsible for some of the differences. For example, taking the case of the collimators following the leaves, with the fields closing in parallel to the direction of leaf motion [see Fig. 1(a)], the equivalent square for the final 2 cm×10 cm segment is small due to the narrow width of the segment. The planning system therefore uses a small output factor. However, in the Elekta linear accelerator, the MLC leaves and the Y backup collimators contribute relatively little to the collimator scatter, and most of the collimator scatter originates from the X‐collimators because these are much closer to the source. Thus, since the 2 cm×10 cm segment has a length of 10 cm, the field behaves like a 10 cm×10 cm field with respect to collimator scatter, and Pinnacle^3^ therefore underestimates the output factor. This can be seen as a slight underestimation in calculated dose (see Table 2). Similar observations can also be made for the other situations considered.

When the complete process of inverse planning and segmentation, together with dose calculation, is tested in the case of orthogonal fields (see section D), the agreement between Pinnacle^3^ and measurements is very promising. The planning system is seen to predict doses that are within about 4% of both the film and ionization chamber measurements.

The verification using the anthropomorphic phantom also shows acceptable results. The TLD study (Fig. 8) shows the predicted dose distribution to be within 4% of the measurements, once outlying measurements have been excluded. This is realistic, given that the treatment plan is for a simulated lung case, where there is considerable tissue inhomogeneity both in and around the PTV. In the film study [Figs. 9(a)) and 9(c)], the agreement between predicted and measured dose is not quite so good, although within the experimental uncertainty, given the difficulties of ensuring minimal air gaps around the film.

Figs. 9(b)) and 9(d) provide an estimation of how these results compare with nonmodulated conformal radiotherapy, with which physicists are more experienced. By verifying the conformal plan, for the same phantom, PTV, and beam directions as the IMRT plan, an indication is obtained of the agreement between treatment planning system and measurements for a much more familiar situation. Note that Pinnacle^3^ has already been commissioned for conformal radiotherapy at this center, the commissioning having included the verification, in several ways, of thoracic treatment plans.^(28)^ In the present study, the correspondence of calculated dose to measured dose is similar for both the IMRT plan and the conformal plan, indicating that a comparable accuracy is being achieved with IMRT as for conformal radiotherapy.

These considerations inevitably lead to the question, What is an acceptable IMRT plan? The Task Group 53 (TG53) report on the accuracy of treatment planning by the American Association of Physicists in Medicine (AAPM) does not formally provide for IMRT.^(31)^ The more recent guidance document by the IMRT subcommittee of the AAPM avoids making a recommendation, but indicates that the growing consensus is that accuracy for IMRT should be comparable to conformal treatments.^(17)^ Taking the closest available situations within TG53 as a guide, it is the collective expectation of members of the task group that the agreement between calculations and measurements may be 3% within the beam and outside of the beam for asymmetric fields, 3% within the beam and 5% outside of the beam for MLC‐shaped fields, and 7% within the beam and outside of the beam for fields in heterogeneous media. The asymmetric and MLC fields may have a penumbra uncertainty of 2 mm to 3 mm, and the fields in heterogeneous media may have a penumbra uncertainty of 7 mm. These figures include the uncertainty associated with the measurements. Similar conclusions are reached by Van Dyk et al.,^(32)^ Venselaar et al.,^(33)^ and Gifford et al.^(34)^

Taking all of these data as a guideline, for the case of IMRT, which consists of a superposition of irregular, asymmetric MLC‐shaped fields in a heterogeneous medium, it is reasonable to expect that the standard deviation of the difference between calculations and measurements may be in the order of 4% in regions of low dose‐gradient and 4 mm in regions of high dose‐gradient. The greatest differences may be two standard deviations away from agreement, in which case correspondingly larger differences may be observed. These estimates are consistent with radiobiological calculations by Mijnheer et al., which show that an uncertainty of less than 3.5% standard deviation should be sought.^(35)^ Practically, the comparisons in Figs. 9(b)) and 9(d) indicate that the IMRT dose distributions generated in this study are comparable in accuracy to that of a typical conformal treatment plan.

## V. CONCLUSIONS

A series of tests has been presented to assess, first, the reliability of an inverse planning algorithm and second, the accuracy of the resulting calculated dose distributions. These tests can form the basis for commissioning an IMRT treatment planning system. In the case of the Pinnacle^3^ system considered, addition of fields to inverse plans mostly results in a reduction of objective function, indicating consistency of inverse planning solutions. Planned dose for fields with stepped intensity agrees with ionization chamber measurements to within 5%. For orthogonal fields, planned dose distributions agree well with dose measured using film and agree with ionization chamber measurements to within 3%. For the anthropomorphic phantom, the standard deviation of difference between planned and measured dose is 4%. These results are in accord with published guidelines and comparable to the accuracy of conformal plans.

## ACKNOWLEDGMENTS

This work has been funded by Cancer Research UK (program grant reference SP2312/0201) and by Philips Radiation Oncology Systems, to whom we are grateful.
